# Low detectable postpartum viral load is associated with HIV transmission in Malawi's prevention of mother‐to‐child transmission programme

**DOI:** 10.1002/jia2.25290

**Published:** 2019-06-10

**Authors:** Megan Landes, Monique van Lettow, Ernest Nkhoma, Beth Tippett Barr, Zinenani Truwah, Erik Shouten, Andreas Jahn, Andrew Auld, Thokozani Kalua, Joep J van Oosterhout

**Affiliations:** ^1^ Dignitas International Zomba Malawi; ^2^ Department of Family and Community Medicine University of Toronto Toronto Canada; ^3^ Dalla Lana School of Public Health University of Toronto Toronto Canada; ^4^ Management Sciences for Health Lilongwe Malawi; ^5^ Centers for Disease Control and Prevention Harare Zimbabwe; ^6^ Ministry of Health Lilongwe Malawi; ^7^ Centers for Disease Control and Prevention Lilongwe Malawi; ^8^ Department of Medicine College of Medicine Blantyre Malawi

**Keywords:** viral load suppression, HIV, prevention of maternal to child transmission, antiretroviral therapy, HIV transmission, Option B+

## Abstract

**Introduction:**

In 2011, Malawi implemented “Option B+,” a test‐and‐treat strategy for the prevention of maternal to child transmission of HIV (PMTCT); however limited data on viral load (VL) suppression exist. We describe VL suppression in HIV‐infected women at four to twenty‐six weeks postpartum, factors associated with VL suppression and the impact of VL suppression levels on MTCT.

**Methods:**

HIV‐positive mothers at four to twenty‐six weeks postpartum were enrolled in a nested cross‐sectional study within the “National Evaluation of Malawi's PMTCT Programme” cohort study between October 2014 and May 2016. HIV‐exposed infants received HIV‐1 DNA testing and venous samples determined maternal VL, classified as unsuppressed (>1000 copies/mL), low‐detectable (40 to 1000 copies/mL) or undetectable (<40 copies/mL). Socio‐demographic and PMTCT indicators were collected. Suboptimal adherence was defined as self‐reported ≥2 days missed ART in the month prior to visit.

**Results:**

Of the 1274 women, 1191 (93.5%) knew their HIV status and 1154/1191 (96.9%) were on ART. VL was available for 1124/1154 (97.4%) of women on ART: 988/1124 (87.9%) had VL suppression of whom 86 (8.7%) had low‐detectable and 902 (91.3%) undetectable VL. Suboptimal adherence was associated with unsuppressed VL (vs. suppressed VL; aOR 3.1, 95% CI 2.0 to 4.9; *p *<* *0.001). Women with low‐detectable VL were more likely to be adolescent (vs. undetectable VL; aOR 3.0, 95% CI 1.4 to 6.6), on ART <6 months (aOR 4.4, 95% CI 2.3 to 8.6), report suboptimal adherence (aOR 2.1, 95% CI 1.1 to 3.8; *p *=* *0.02), and less likely to have primary or secondary education (vs. none; aOR 0.3, 95% CI 0.2 to 0.7 or aOR 0.3, 95% 0.1 to 0.6). MTCT ratios among women on ART who had undetectable VL, low‐detectable VL and unsuppressed VL were 0.9% (8/902; 95% CI 0.3 to 1.5), 7.0% (6/86; 95% CI 1.5 to 12.5) and 14.0% (19/136; 95% CI 8.1 to 20.0). Unsuppressed VL and low‐detectable VL (vs. undetectable VL) increased the risk of MTCT 17‐fold (aOR 17.4, 95% CI 7.4 to 41.1; *p *=* *0.002) and ninefold (aOR 8.5, 95% CI 2.9 to 25.2; *p *<* *0.001).

**Conclusions:**

Unsuppressed and low‐detectable VL was strongly predictive of MTCT among women on ART and associated with suboptimal adherence. This urges further consideration of optimal VL monitoring and target levels to reach elimination of paediatric infection.

## 1 Introduction

In 2011, Malawi implemented “Option B+,” a universal test and treat strategy for all pregnant and breastfeeding women for the prevention of mother‐to‐child‐transmission (PMTCT) of HIV 1, 2. Option B+ has led to markedly increased rates of antiretroviral therapy (ART) initiation of among HIV‐infected women of reproductive age 3, 4. However, little is known about the degree to which women on ART achieve sustained viral load (VL) suppression during pregnancy, delivery and breastfeeding, which is a key determinant of mother‐to‐child‐transmission (MTCT).

Since 2015, routine VL monitoring in Malawi has been challenged by human resource shortages, difficulties in sample transportation to centralized laboratories, and laboratory capacity 5. Furthermore, national ART programme VL suppression estimates do not accurately capture women in Option B+ as VL testing is rarely performed during pregnancy or breastfeeding 4. Regionally, countries face similar challenges in estimating VL suppression in the Option B+ population and there is a lack of consensus on best practices of VL monitoring for women initiating ART during pregnancy or breastfeeding 6. Extrapolating from study settings, VL suppression estimates are 84.6% among women enrolled in Option B+ during late pregnancy/early postpartum in Rwanda and 89.6% in Uganda among women starting ART in pregnancy and retained in care at five years 7, 8.

Further complicating an understanding of how VL suppression impacts MTCT within Option B+ is the variation in VL suppression definitions throughout the region. In Malawi, VL suppression is defined as <1000 copies/mL 9, yet a relevant proportion of MTCT occurred in women enrolled in Option B+ in South Africa with low‐detectable VL levels, that is, between 40 and 1000 copies/mL 10. Few PMTCT programmes, including the Malawi HIV programme, distinguish between low‐detectable and undetectable VL levels.

The National Evaluation of Malawi's PMTCT Programme (NEMAPP) study launched in 2014 to evaluate the effectiveness of Option B+ by enrolling HIV‐infected women and their infants at four to twenty‐six weeks postpartum and following them for 24 months. Within this nationally representative cohort, uptake of PMTCT services was very high: 97.8% of women knew their HIV status at enrolment and 96.3% of these were on ART 11. Here, we describe VL suppression rates, factors associated with VL suppression and MTCT ratios stratified by low‐detectable, undetectable and unsuppressed VL, at four to twenty‐six weeks postpartum among a sample of women enrolled in the NEMAPP study. These findings are relevant for Malawi's performance against the UNAIDS 90‐90‐90 targets by providing insight into the third “90” among pregnant and breastfeeding women on ART 12.

## 2 Methods

**Figure 1:**
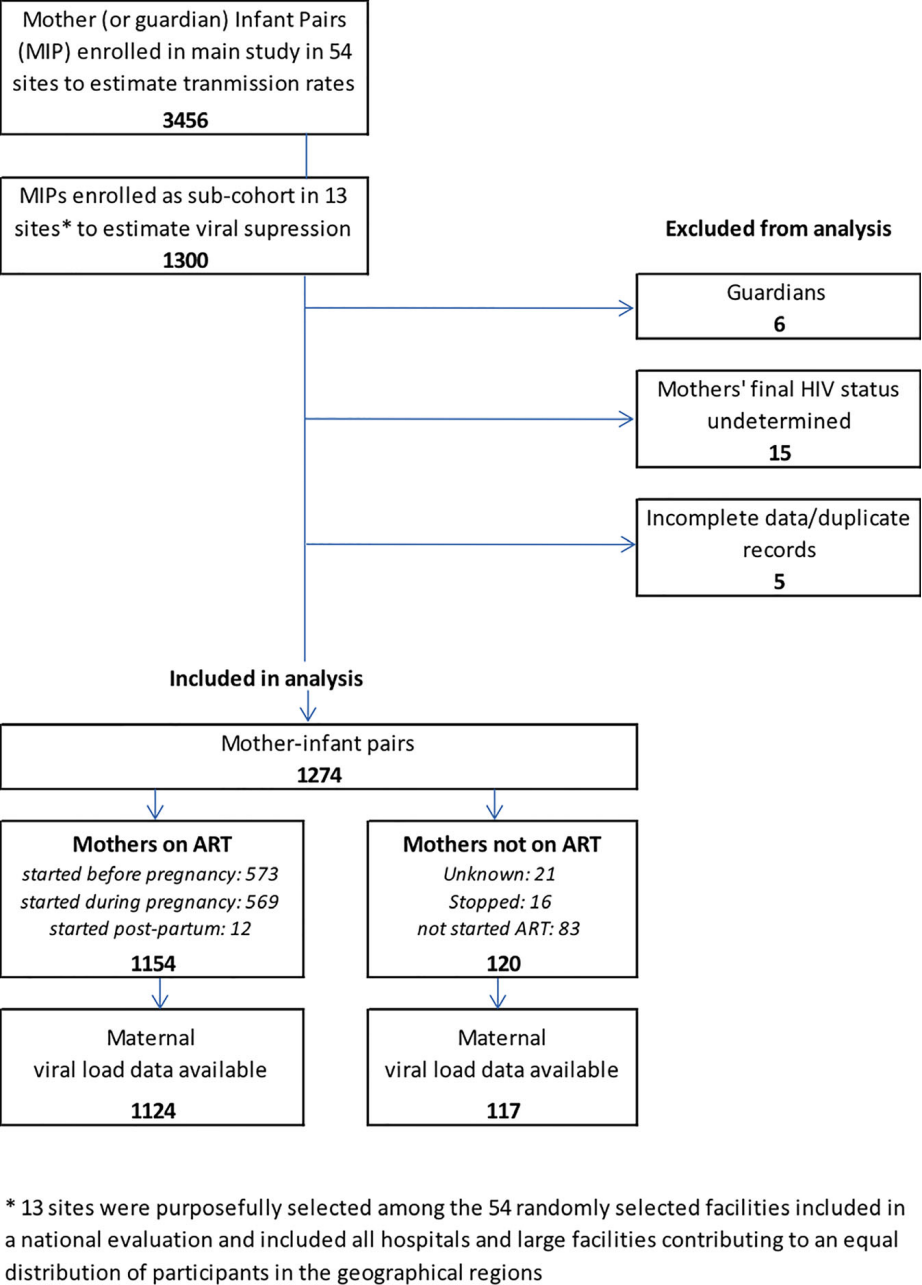
Flow chart of mother‐infant pairs excluded and included in the study.

This is a nested cross‐sectional study of HIV‐infected mothers presenting with their four to twenty‐six week old infants at outpatient clinics who were enrolled in longitudinal follow‐up in the National Evaluation of the Malawi PMTCT Programme (NEMAPP) study between October 2014 and May 2016. NEMAPP used a multistage cluster design to sample 54 sites across Malawi 13 and this sub‐set was derived using probability proportionate‐to‐size sampling methods to select 13 sites across the eight districts from the original sites. Women in these selected sites were simultaneously consented for enrolment in both the main study and in this subset for intensive clinical and laboratory monitoring. All mother‐infant pairs were followed up at 12 and 24 months. The study period occurred three years after the national implementation of “Option B+” PMTCT guidelines in Malawi which provided lifelong ART (i.e. tenofovir/lamivudine/efavirenz) for all pregnant and breastfeeding women. Mothers (or guardians) with four to twenty‐six week infants were screened for HIV infection by the study team and 3456 HIV‐positive mothers (or guardians) with HIV‐exposed infants were consented for enrolment in the main study. A sample of 1324 HIV‐positive mothers was calculated to estimate VL suppression based on an estimated 50% suppression rate, 50% loss to follow‐up at 24 months and a precision of 2.5% with a 95% confidence interval (95% CI) and an assumed design effect of 2.0. Guardians were excluded from the current analysis (Figure 1).

Included mothers were interviewed using structured questionnaires by trained health facility staff to obtain information about age, parity, education, HIV status at screening, uptake PMTCT/ART, adherence to treatment (self‐reported number of days missed ARVs in the last month), birthweight of infant, uptake of infant nevirapine prophylaxis and breastfeeding practices. When possible, mothers’ health booklets were checked for accuracy of responses.

A qualitative HIV‐1 DNA polymerase chain reaction (COBAS AmpliPrep/COBAS TaqMan Qualitative Assay Version 2.0, detection level 221 copies/mL; Roche Diagnostics, USA) test was performed on all HIV‐exposed infant dried blood spot (DBS) samples to determine HIV infection at the time of enrolment into NEMAPP (as part of study procedures and outside of national PMTCT programme testing). Within this subset, maternal HIV viral load (VL) testing was conducted at enrolment on venous samples (Abbott Real‐Time HIV‐1 Assay, Abbott Laboratories, Chicago, IL) of all women regardless of ART status. VL suppression is defined as HIV 1‐RNA <1000 copies/mL as per the Malawi national HIV guidelines 1. We further categorized VL results as “undetectable” (<40 copies/mL), “low‐detectable” (40 to 1000 copies/mL) and “unsuppressed” (>1000 copies/mL), (with “suppressed VL” being the inverse, i.e. all women with <1000 copies/mL).

Missing data were treated as additional categories. Crude percentages were calculated and comparisons between groups made using chi‐square tests for categorical variables and non‐parametric tests for medians, using normal approximations (Wald) methods to calculate confidence intervals. Multivariable logistic regression analysis was used to identify characteristics associated with unsuppressed versus suppressed VL, with low‐detectable versus undetectable VL, and with MTCT. Univariate odds ratios (OR) with 95% CI were calculated for each variable in the model using normal approximation (Wald) methods. Adjusted OR (aOR) with 95% CI were calculated for each model after adjustment for age, parity, education, time on ART, number of days missed ARV's in last month and exposure to PMTCT in a previous pregnancy. In addition, birthweight, uptake of infant nevirapine, exclusive breastfeeding and VL categories were included in the model for MTCT. All variables were simultaneously entered in the logistic regression model and tested for removal through backward stepwise selection. A 0.05 significance level was set for all statistical testing. Analyses were conducted using IBM SPSS Statistics 24 (IBM, Armonk, NY, USA).

Ethical approval was received from Malawi's National Health Sciences Research Committee (#1262) and the University of Toronto (#30448). The US Centers for Disease Control and Prevention (CDC) reviewed and approved as research according to human research protection procedures (#2014‐054‐7), but was not engaged. All participants provided written informed consent.

The funding source for this study was the U.S. President's Emergency Plan for AIDS Relief (PEPFAR) and they had no direct role in study design, implementation, analysis or preparation/submission of this manuscript. The author acknowledges full access to all the data and final responsibility for submission.

## 3 Results

A total of 1274 mother‐infant pairs were included in this study (see Figure 1). Table 1 describes the characteristics of mothers: median age was 29 years (IQR 23 to 33) and median parity was three births (IQR 2 to 4). The majority had no formal education or primary school only (836/1274; 65.6%).

**Table 1:** Characteristics of women included in the study

	n	n = 1274
Mother age in years, median (IQR)	1272	29 (23 to 33)
Mother's age in years, %		
≤19	86	6.8
20 to 24	284	22.3
25 to 29	338	26.5
≥30	564	44.3
Missing	2	0.2
Infant age at enrolment in months, median (IQR)	1274	1.8 (1.5 to 3.1)
Parity, median (IQR)	1272	3 (2 to 4)
Parity, %		
1	189	14.8
2 to 3	630	49.5
≥4	453	35.6
Missing	2	0.2
Level of education, %		
None	114	8.9
Primary education	722	56.7
Secondary education	416	32.7
Post‐secondary education	22	1.7
Maternal HIV status at time of study screening, % (four to twenty‐six weeks postpartum)		
Already known HIV‐infected	1191	93.5
Newly diagnosed HIV‐infected	83	6.5
Exposure to PMTCT in a previous pregnancy		
None	734	57.6
Short course PMTCT before Option B+ (sdNVP or AZT)	123	9.7
Triple ART (for own health or option B+)^a^	396	31.1
Missing	21	1.6
Maternal ART Status, %		
On ART (started before last pregnancy)	573	45.0
On ART (started during last pregnancy)	569	44.7
On ART (started ART postpartum)	12	0.9
Started but Stopped ART	16	1.3
Unknown, did not want to reveal	21	1.6
Did not start ART yet (newly diagnosed)	83	6.5
Time on ART in months (at time of study) among those on ART, median (IQR)	940	9.9 (5.7 to 44.1)
Time on ART in months (at time of study) among those on ART, %		
≤2.0	36	3.1
2.1 to 6.0	225	19.5
6.1 to 12.0	227	19.7
12.1 to 18.0	38	3.3
18.1 to 24.0	42	3.6
≥24	372	32.2
Missing	214	18.5
Number of days having missed ART in the last month, among those on ART		
0	910	78.9
1 day	93	8.1
≥2 days	136	11.8
Missing	15	1.3
Birthweight of index infant		
Low birthweight, 1.5 to 2.5 kg	235	18.4
Normal birthweight, 2.5 to 4.2 kg	993	77.9
Missing	46	3.6
Infant nevirapine prophylaxis received by exposed infants		
Received nevirapine	1161	91.1
Did not receive nevirapine	113	8.9
Exclusive breastfeeding (reported no other food)		
Yes	1164	91.4
No	108	8.5
Missing	2	0.2

a Started prior to or during previous pregnancy, independent of current ART status.

Overall, 1191 (93.5%) women knew their HIV status at enrolment and 1154 (96.9%) of these women were on ART. Among women on ART, 573 (49.7%) started ART before and 569 (49.3%) during the index pregnancy, while 12 (1.0%) started postpartum. The median duration on ART for those starting before pregnancy, during pregnancy and postpartum was 39.3 months (IQR 18.3 to 59.0), 6.3 months (4.6 to 8.5) and 1.2 months (0.5 to 2.9) respectively (See Table 2). Among women on ART, 910 (78.9%) reported missing no days of ART in the last month.

**Table 2:** Antiretroviral treatment (ART) and viral load

	Total	Viral load available	Duration on ART in months	Median viral load	Viral Load <40	Viral load 40 to 1000	Viral load >1000
	n	n	Median (IQR)	(IQR)	% (95% CI)	% (95% CI)	% (95% CI)
On ART (started before index pregnancy)	573	556	39.3 (18.3 to 59.0)	<40	84.4 (81.3 to 87.4)	4.5 (2.8 to 6.2)	11.1 (8.5 to 13.8)
On ART (started during index pregnancy)	569	556	6.3 (4.6 to 8.5)	<40	76.3 (72.7 to 79.8)	10.4 (7.9 to 13.0)	13.3 (10.5 to 16.1)
On ART (started ART postpartum)	12	12	1.2 (0.5 to 2.9)	<40 (<40 to 194)	75.0 (46.3 to 99.9)	25.0 (0.1 to 53.7)	‐
Not on ART	120	117	‐	16,016 (1125 to 57,235)	12.0 (6.0 to 17.9)	12.0 (6.0 to 17.9)	76.1 (68.2 to 83.9)
Total	1274	1241	9.9 (5.7 to 44.1)	<40 (<40 to 58)	73.8 (71.4 to 76.3)	8.1 (6.5 to 9.6)	18.1 (16.0 to 20.3)

Among the 120 women who reported not being on ART at enrolment, 16 (13.3%) had started but stopped ART, 21 (17.5%) did not wish to disclose their HIV status to the study team prior to HIV testing during screening and 83 women (69.2%) were newly diagnosed HIV infections (i.e. either HIV negative or unknown prior to testing HIV positive at enrolment).

Of the 1274 infants born, 235 (18.4%) were low birthweight (<2.5Kg), of which 7 (3.0%) were very low birthweight (<1.5 kg). 91.1% of infants received nevirapine prophylaxis and 91.4% were exclusively breastfed until enrolment.

VL results were available for 1241 (97.4%) women: 1124/1154 (97.4%) of women on ART and 117/120 (97.5%) of women not on ART (see Table 2). Among women on ART with available VL (N = 1124), 988 (87.9%) achieved VL suppression, of whom 902 (91.3%) had undetectable VL and 86 (8.7%) had low‐detectable VL.

Achieving undetectable VL occurred more in women who started ART prior to pregnancy compared to during pregnancy (469/556: 84.4% vs. 424/556: 76.3%; *p *=* *0.001). Conversely, having low‐detectable VL occurred more in women who started ART during pregnancy compared to those starting before (58/556:10.4% vs. 25/556: 4.5%; *p *<* *0.001). Among women who started ART postpartum, 100% (12/12) achieved VL suppression and 9/12 (75%) achieved undetectable VL. There was no significant difference in the proportion of women with unsuppressed VL between those who started ART prior to this pregnancy and those who started during the pregnancy (62/556: 11.2% vs. 74/556: 13.3%; *p *=* *0.27).

The majority of women not on ART had unsuppressed VL (89/117: 76.1%) and had significantly higher median VL than women on ART (see Table 2; *p *=* *0.001). The majority of women not on ART and with unsuppressed VL were those with newly diagnosed infections (67/89; 75.3%) at the time of enrolment. Of the 28 (24.0%) women not on ART but who had either suppressed or low‐detectable VL, 11 (39.3%) did not reveal their HIV status (or ART use) to the study team at enrolment and 4 (14.3%) had started or stopped ART (date of stopping not available).

Table 3 describes MTCT for women on ART and those not on ART stratified by viral load categories. Of the 34 transmissions occurring among women on ART, 6 (17.6%) occurred in women with low detectable VL and 19 (55.9%) occurred in women with unsuppressed VL. MTCT ratios among women on ART with undetectable VL, low detectable VL and unsuppressed VL were 0.9% (8/902; 95% CI 0.3 to 1.5), 7.0% (6/86; 95% CI 1.6 to 12.4) and 14.0% (19/136; 95% CI 8.1 to 19.8) respectively.

**Table 3:** MTCT at four to twenty‐six weeks postpartum, stratified by ART status^a^ and Viral Load

	On ART				Not on ART				
	Median VL		MTCT		Median VL		MTCT		Chi‐square
	Total	(IQR)	n	% (95% CI)	Total	(IQR)	n	% (95% CI)	*p*‐value^b^
All	1154	<40	34	3.0 (2.0 to 3.9)	120	16,016 (1125 to 57,235)	32	26.7 (18.8 to 34.6)	<0.001
VL < 40	902	<40	8	0.9 (0.3 to 1.5)	14	<40	2	14.3 (0.0 to 32.6)	<0.001
VL 40 to 1000	86	168 (68 to 382)	6	7.0 (1.6 to 12.4)	14	420 (134 to 576)	3	21.4 (0.0 to 42.9)	0.08
VL > 1000	136	11,618 (3821 to 44,815)	19	14.0 (8.1 to 19.8)	89	34,349 (9826 to 83,203)	27	30.3 (20.8 to 39.9)	0.003
Missing VL result	30		1		3		‐		

^a^Maternal self‐report at enrolment; ^b^for the comparison of MTCT ratios between groups.

Among women not on ART, MTCT ratios for those with undetectable VL, low detectable VL and unsuppressed VL were 14.3% (2/14; 95% CI 0.0 to 32.6), 21.4% (3/14; 95% CI 0.0 to 42.9) and 30.3% (27/89; 95% CI 20.8 to 39.9) respectively. Among the 32 infections occurring in women not on ART, 24 (75%) occurred among women with new infections at the time of study (1 had low‐detectable virus and 20 (83.3%) had unsuppressed VL) and the MTCT rate among new infections overall was 24/83 (28.9%). There were significant differences in both median VL and MTCT between women on ART and those not on ART with unsuppressed VL (median VL 11,618 vs. 34,349, *p *=* *0.005 and 14.0 vs. 30.3, *p *=* *0.003 respectively).

In multivariable analysis, women with unsuppressed VL were more likely than women with suppressed VL to report suboptimal adherence (aOR 3.1, 95% CI 2.0 to 4.9; *p *<* *0.001) when adjusted for age, parity, education, time on ART and previous exposure to PMTCT/ART. Women with low‐detectable VL were more likely than those with undetectable VL to be adolescent (aOR 3.0, 95% CI 1.4 to 6.6), on ART for less than six months (aOR 4.4, 95% CI 2.3 to 8.6) and have suboptimal adherence (aOR 2.1, 95% CI 1.1 to 3.8; *p *=* *0.02), and less likely to have primary or secondary education versus no education (aOR 0.3, 95% CI 0.2 to 0.7 and aOR 0.3, 95% 0.1 to 0.6 respectively; see Table 4).

**Table 4:** Factors associated with unsuppressed (>1000) versus suppressed (<1000) and low‐detectable (VL 40 to 1000) versus undetectable (<40) maternal viral load among Malawian women on ART four to twenty‐six weeks postpartum (for whom VL available)

	Unsuppressed (>1000) vs. suppressed (<1000) VL						Low‐detectable (40 to 1000) vs. undetectable (<40) VL					
	n/n	%	Univariate (unadjusted)		Multivariable (adjusted)		n/n	%	Univariate (unadjusted)		Multivariable (adjusted)	
			OR (95% CI)	*p*‐value	aOR (95% CI)^a^	*p*‐value			OR (95% CI)	*p*‐value	aOR (95% CI)^a^	*p*‐value
Total	136/1124	12					86/988	9				
Mother's age in years												
≤19	9/72	13	1.1 (0.5 to 2.4)	0.72			12/63	19	3.7 (1.8 to 7.7)	<0.001	3.0 (1.4 to 6.6)	0.006
20 to 24	36/230	16	1.5(1.0 to 2.3)	0.08			19/194	10	1.7 (0.9 to 3.1)	0.09	1.6 (0.8 to 3.0)	0.15
25 to 29	33/297	11	1.0 (0.6 to 1.6)	0.99			27/264	10	1.8 (1.0 to 3.1)	0.04	2.0 (1.0 to 3.5)	0.05
≥30	58/523	11	ref				28/465	6	ref		ref	
Missing	0/2	0										
Parity												
1	22/159	14	1.3 (0.8 to 2.2)	0.34			14/137	10	1.7 (0.9 to 3.5)	0.12		
2 to 3	68/544	13	1.2 (0.8 to 1.7)	0.47			49/476	10	1.7 (1.0 to 2.9)	0.03		
≥4	46/419	11	ref				23/373	6	ref			
Missing	0/2	0					0/2	0				
Level of education												
None	9/100	9	ref				16/91	18	ref		ref	
Primary education	84/632	13	1.6 (0.8 to 3.2)	0.23			44/548	8	0.4 (0.2 to 0.8)	0.005	0.3 (0.2 to 0.7)	0.002
Secondary or post‐secondary education	43/392	11	1.2 (0.6 to 2.6)	0.57			26/349	7	0.4 (0.2 to 0.7)	0.004	0.3 (0.1 to 0.6)	0.001
Time on ART (months)												
<6	22/254	9	0.7 (0.4 to 1.3)	0.29	0.7 (0.4 to 1.3)	0.29	39/232	17	4.4 (2.4 to 8.4)	<0.001	4.4 (2.3 to 8.6)	<0.001
6 to 12	25/221	11	1.0 (0.6 to 1.7)	0.99	0.9 (0.5 to 1.5)	0.63	14/196	7	1.7 (0.8‐3.6)	0.18	1.4 (0.7 to 3.1)	0.36
12 to 24	8/79	10	0.9 (0.4 to 2.0)	0.77	0.9 (0.4 to 2.1)	0.86	2/71	3	0.6 (0.1 to 2.9)	0.56	0.7 (0.2 to 3.1)	0.63
>24	41/363	11	ref		ref		14/322	4	ref		ref	
Missing	40/207	19	1.9 (1.2 to 3.0)	0.009	1.7 (1.0 to 2.8)	0.02	17/167	10	2.5 (1.2 to 5.2)	0.02	2.5 (1.2 to 5.4)	0.02
No. of days having missed ARVs in the last month												
0	89/886	10	ref		ref		68/797	9	ref		ref	
1 day	8/90	9	0.9 (0.4 to 1.9)	0.73	0.9 (0.4 to 2.0)	0.82	1/82	1	0.1 (0.0 to 1.0)	0.05	0.1 (0.1 to 1.0)	0.07
≥2 days	35/134	26	3.2 (2.0 to 4.9)	<0.001	3.1 (2.0 to 4.9)	<0.001	17/99	17	2.2 (1.2 to 4.0)	0.07	2.1 (1.1 to 3.8)	0.02
Missing	4/14	29	3.6 (1.1 to 11.7)	0.03	2.9 (0.9 to 9.7)	0.08	0/10					
Exposure to PMTCT in a previous pregnancy												
None	71/611	12	ref				57/540	11	ref			
Short course PMTCT before Option B+ (sdNVP or AZT)	13/120	11	0.9 (0.5 to 1.7)	0.81			5/107	5	0.4 (0.2 to 1.1)	0.07		
Triple ART (for own health or option B+)^b^	51/373	14	1.2 (0.8 to 1.8)	0.34			23/322	7	0.7 (0.4 to 1.1)	0.09		
Missing	1/20	5	0.4 (0.1 to 3.0)	0.38			1/19	5	0.5 (0.1 to 3.6)	0.47		

All variables were simultaneously entered in the logistic regression model as the first step and tested for removal one by one. In the multivariable analysis, only variables with significant associations in the last step are shown.

^a^Controlled for all other variables shown in this table; ^b^started prior to or during previous pregnancy, independent of current ART status.

Table 5 shows multivariable analysis of characteristics associated with MTCT among women on ART. Compared to having undetectable VL, unsuppressed VL and low‐detectable VL increased the odds of MTCT 17‐fold (aOR 17.4, 95% CI 7.4 to 41.1; *p *=* *0.002) and ninefold (aOR 8.5, 95% CI 2.9 to 25.2; *p *<* *0.001).

**Table 5:** Factors associated with MTCT among Malawian women on ART

All	n = 34/1154	2.9%	Univariate (unadjusted)		Multivariable (adjusted)	
			OR (95% CI)	*p‐*value	aOR (95% CI)^a^	*p*‐value
Mother's age in years						
≤19	4/74	5.4%	2.7 (0.8 to 8.8)	0.09		
20 to 24	8/234	3.4%	1.7 (0.7 to 4.2)	0.27		
25 to 29	11/309	3.6%	1.8 (0.8 to 4.1)	0.19		
≥30	11/535	2.1%	ref			
Missing	0/2	0%				
Parity						
1	5/163	3.1%	1.2 (0.4 to 3.5)	0.74		
2 to 3	18/560	3.2%	1.3 (0.6 to 2.7)	0.55		
≥4	11/429	2.6%	ref			
Missing	0/2	0%				
Level of education						
None	3/103	2.9%	ref			
Primary education	23/650	3.5%	1.2 (0.4 to 4.1)	0.75		
Secondary education	8/379	2.1%	0.7 (0.2 to 2.8)	0.63		
Post‐secondary education	0/22	0%				
Time on ART (months)						
<6	10/261	3.8%	2.4 (0.9 to 6.8)	0.09		
6 to 12	10/227	4.4%	2.8 (1.0 to 7.8)	0.05		
12 to 24	2/80	2.5%	1.6 (0.3 to 7.9)	0.59		
>24	6/372	1.6%	ref			
Missing	6/214	2.8%	1.8 (0.6 to 5.5)	0.33		
Nr of days having missed ART in the last month						
0	25/910	2.7%	ref			
1 day	3/93	3.2%	1.2 (0.3 to 4.0)	0.79		
≥2 days	5/136	3.7%	1.4 (0.5 to 3.6)	0.55		
Missing	1/15	6.7%	2.5 (0.3 to 20.0)	0.38		
Birthweight of index infant						
Low birthweight, 1.5 to 2.5 kg	7/203	3.4%	1.2 (0.5 to 2.8)	0.65		
Normal birthweight, 2.5 to 4.2 kg	26/912	2.9%	ref			
Missing	1/39	2.6%	0.9 (0.1 to 6.8)	0.91		
Infant nevirapine prophylaxis received by exposed infants						
Received nevirapine	32/1118	2.9%	ref			
Did not receive nevirapine	2/36	5.6%	2.0 (0.5 to 8.7)	0.36		
Exclusive breastfeeding (reported no other food)						
Yes	27/1064	2.5%	ref			
No	6/88	6.8%	2.8 (1.1 to 7.0)	0.03		
Missing	1/2	50%	38.4 (2.3 to 630.3)	0.01		
Previous exposure to PMTCT						
No previous exposure to ART/PMTCT)	22/628	3.5%	ref			
Previous short course (sdNVP or AZT) for PMTCT	5/121	4.1%	1.2 (0.4 to 3.2)	0.73		
Previous triple ART prior to pregnancy	7/384	1.8%	0.5 (0.2 to 1.2)	0.13		
Missing	0/21	0%				
Viral load among all						
<40	8/902	0.9%	ref			
40 to 1000	6/86	7.0%	8.4 (2.8 to 24.8)	<0.001	8.5 (2.9 to 25.2)	<0.001
>1000	19/136	14.0%	18.1 (7.8 to 42.4)	<0.001	17.4 (7.4 to 41.1)	0.002
Missing	1/30	3.3%	3.9 (0.5 to 31.8)	0.21	3.4 (0.4 to 28.2)	0.27

All variables were simultaneously entered in the logistic regression model as the first step and tested for removal one by one.

In the multivariable analysis, only variables with significant associations in the last step are shown.

a Controlled for all other variables shown in this table.

## 4 Discussion

In this nationally representative cohort of HIV‐infected women enrolled in Malawi's PMTCT programme, the postpartum viral load (VL) suppression ratio for women on ART is approaching the third UNAIDS 90‐90‐90 goal of 90% VL suppression. While not being on ART was associated with detectable VL and MTCT, among those on ART, unsuppressed VL and low‐detectable VL remained the strongest predictor of transmission. Almost 9% of women traditionally classified as reaching VL suppression (<1000 copies/mL) in fact had low‐detectable levels of viraemia (40 to 1000 copies) and accounted for more than one in six of all MTCT infections occurring in women on ART. Suboptimal adherence was the strongest predictor of both low‐detectable VL and unsuppressed VL, along with adolescent age, lower levels of education and less time on ART.

Our VL suppression estimate indicates that the Malawi PMTCT programme is close to reaching the final UNAIDS goal of 90% among HIV‐infected pregnant and breastfeeding women on ART. Similar estimates are documented in the postpartum period in three Option B+ study cohorts: 81.2% among Zimbabwean women at four to twelve weeks 14, 84% among Malawian women at six months postpartum 15 and 84% among Rwandan women in late pregnancy/early postpartum 7. Among women who achieve VL suppression, the Malawi PMTCT programme shows remarkable reductions in MTCT. This impact is greatest among women on ART reaching undetectable VL where the MTCT ratio (i.e. <1%) approaches estimates in higher resource settings 16, 17.

However, we identify a strong association between any detectable VL in the early postpartum period and MTCT: women on ART with unsuppressed VL had a 17‐fold increased risk of MTCT and those with low‐detectable VL had a ninefold increased risk. Notably, almost 9% of women traditionally classified by WHO guidelines as suppressed VL (i.e. <1000 copies/mL) had a low‐detectable VL and accounted for almost 20% of all transmissions. Limited data exist on the role of low level viraemia among women on ART during pregnancy, delivery or breastfeeding in MTCT risk in the region, however one recent South African cohort of women initiated on ART during pregnancy demonstrated an increased risk of MTCT for women with low detectable VL versus those with undetectable VL (<50 copies/mL; 2 vs. 0.2%) at eight weeks of age 10. We estimate that in Malawi, women with low‐detectable viraemia (vs. undetectable VL) on ART in the early postpartum period contribute an excess 460 new infant HIV infections yearly (i.e. 60 infections/100,000 births per annum) 18. These excess infections alone place Malawi above the level of control as outlined by the WHO (50 per 100,000 live births) for the elimination of MTCT and highlights the high priority of identifying detectable VL levels during pregnancy, delivery and breastfeeding 19. Challenges exist to determining low‐detectable levels in much of the region, and currently in Malawi most VL tests are done using DBS, which has a viral load detection limit of 1000 copies/mL. These data contribute to an urgent consideration of VL target levels to eliminate MTCT in this region.

While we cannot comment on the trajectory of VL levels for women on ART with low‐detectable or unsuppressed virus during pregnancy and delivery, our findings contribute to regional discussions regarding the impact that VL monitoring in pregnancy/breastfeeding could have on improving MTCT rates 6, 20. Currently in the region, VL monitoring is rarely coordinated with delivery in pregnant women living with HIV and in Malawi guidelines still recommended VL testing at six months after ART initiation and then every two years 1. In routine ART programmes, VL monitoring and suppression “thresholds” trigger a cascade of interventions by clinicians to improve patient outcomes. These include intensification of efforts to first support adherence and second, after sustained elevated VL, to consider resistance testing to guide switching regimens. The importance of having stratified VL information for clinicians working with women initiating ART in pregnancy and breastfeeding to trigger this cascade of interventions should be considered a priority, especially given the short timelines associated with the PMTCT cascade of HIV detection, ART initiation and ultimately delivery of an infant and exposure to breastfeeding. These findings call for urgent discussion about timing and frequency of VL monitoring for pregnant women.

Here, we found that women on ART with both unsuppressed and low‐detectable VLs were more likely to self‐report suboptimal adherence in the past month which supports recent findings in South Africa, in the context of Option B+, that longitudinal self‐reported adherence could predict episodes of viraemia in pregnant and postpartum women 21. This suggests that self‐report may be useful in this programmatic context to identify women needing intensified adherence interventions to improve VL suppression, even in the absence of routine VL testing during pregnancy. In Malawi, national guidelines state adherence should be ascertained at all clinical appointments, followed by group counselling or intensified adherence support if adherence is inadequate. However, studies of similar populations in the region describe ongoing challenges for women in adherence during pregnancy and breastfeeding 22, 23. While limited evidence exists that links specific adherence interventions to VL suppression targets in this population, some studies have shown promising interventions for adherence interventions such as counselling or peer support mechanisms in the general population 24.

The ideal timing and frequency of VL measurement during pregnancy and breastfeeding also remains unclear, including an understanding of how long it would take to reach undetectable VL with intensified adherence interventions. In a South African cohort of women initiated on ART in pregnancy, time to undetectable VL was 28 days and we similarly report that 100% of women in this cohort starting ART postpartum (median duration 1.2 months) had reached VL suppression and 75% had undetectable VL 10. By increasing VL availability before delivery (i.e. <4 weeks prior) improvements may be made in adherence, VL suppression rates and infant outcomes. Furthermore, consideration could be made for those women who are identified with detectable VL for intensifying infant ART prophylaxis at birth and/or to receive ongoing monitoring and adherence interventions for maintaining VL suppression during breastfeeding, and or to receive resistance testing to guide potential regimen switches.

We additionally note that achieving undetectable VL was more challenging for adolescents and for women who had less education and less time on ART. Regional evidence highlights that adolescents are more likely to miss steps in the PMTCT cascade and suboptimally engage in care, yet studies supporting adherence interventions purposefully targeting pregnant adolescents are extremely limited 11, 25, 26, 27. Interventions to improve adherence, especially those appropriately targeted to age and education level, and or the addition of intensified prophylaxis for infants (PREP) of women with any detectable VL prior to delivery or in the early postpartum period have not yet been assessed but could help inform PMTCT programmes regarding VL monitoring frequency and suppression targets.

## 5 Strengths and limitations

This study has some particular strengths in estimating levels of VL suppression, which include that it is nationally representative and describes a programmatic evaluation of Option B+ in Malawi. The power calculation for this study was done several years ago, in 2012 as Option B+ was scaling up in Malawi, and before we had further regional data to provide more precise estimates of VL suppression. However, the initial underestimation of population‐level VL suppression for this study ultimately improves the final study power. Additionally, we explore the association between stratified, precision VL measures to understand the impact of low‐level viraemia on MTCT. We are limited by VL measures being obtained at four to twenty‐six weeks postpartum, without pre‐ART or pre‐delivery VL measures, and cannot comment on the trajectory of VL individually. Also, we cannot confirm incident infection among the 6.5% of women identified as new infections at screening/enrolment nor are we able to examine the potential role of drug resistance, as one study in Malawi reported a 35% resistance rate to NNRTIs among 55 HIV‐infected pregnant women 15. Finally, small numbers in the stratified VL categories may decrease MTCT accuracy and we did not account for site level clustering as the sample was derived from regional strata.

## 6 Conclusions

In this nationally representative cohort of HIV‐infected women enrolled in Malawi's Option B+ programme, postpartum viral load (VL) suppression for women on ART is approaching the third UNAIDS 90‐90‐90 goal of 90% VL suppression. However, we identify a strong association between any detectable VL in the early postpartum period and a considerable population risk of MTCT. These findings call for urgent discussion about the availability, timing and frequency of VL monitoring in Malawi and whether VL monitoring should be able to detect lower levels of viraemia (i.e. <40 or < 20 copies/mL). Additionally, more research is required that explores optimal timing of VL measurement and effective interventions for achieving VL suppression in this population and the impact on eliminating paediatric HIV.

## Competing interests

The authors declare that they have no competing interests.

## Authors’ contributions

The literature review was conducted by M.L., M.v.L. and E.N. Study design and methods were developed by M.L., M.v.L., E.N., B.T.B., E.S., A.J., J.v.O. and T.K. Data analysis was done by M.L., M.v.L., E.N. and J.v.O. Data interpretation was done by M.L., M.v.L., E.N., B.T.B., Z.T., E.S., A.J., A.A., T.K. and J.v.O. Writing was completed by M.L., M.v.L., E.N. and J.v.O. All authors reviewed the final manuscript for submission.
